# Predictors of “Liking” Three Types of Health and Fitness-Related Content on Social Media: A Cross-Sectional Study

**DOI:** 10.2196/jmir.4803

**Published:** 2015-08-21

**Authors:** Elise R Carrotte, Alyce M Vella, Megan SC Lim

**Affiliations:** ^1^ Centre for Population Health Burnet Institute Melbourne Australia; ^2^ School of Public Health and Preventive Medicine Monash University Melbourne Australia

**Keywords:** fitspiration, social media, blogging, adolescent, physical fitness, eating disorders, women’s health

## Abstract

**Background:**

Adolescence and young adulthood are key periods for developing norms related to health behaviors and body image, and social media can influence these norms. Social media is saturated with content related to dieting, fitness, and health. Health and fitness–related social media content has received significant media attention for often containing objectifying and inaccurate health messages. Limited research has identified problematic features of such content, including stigmatizing language around weight, portraying guilt-related messages regarding food, and praising thinness. However, no research has identified who is “liking” or “following” (ie, consuming) such content.

**Objective:**

This exploratory study aimed to identify demographics, mental health, and substance use–related behaviors that predicted consuming 3 types of health and fitness–related social media content—weight loss/fitness motivation pages (ie, “fitspiration”), detox/cleanse pages, and diet/fitness plan pages—among young social media users.

**Methods:**

Participants (N=1001; age: median 21.06, IQR 17.64-24.64; female: 723/1001, 72.23%) completed a cross-sectional 112-question online survey aimed at social media users aged between 15-29 years residing in Victoria, Australia. Logistic regression was used to determine which characteristics predicted consuming the 3 types of health and fitness–related social media content.

**Results:**

A total of 378 (37.76%) participants reported consuming at least 1 of the 3 types of health and fitness–related social media content: 308 (30.77%) fitspiration pages, 145 (14.49%) detox pages, and 235 (23.48%) diet/fitness plan pages. Of the health and fitness–related social media content consumers, 85.7% (324/378) identified as female and 44.8% (324/723) of all female participants consumed at least 1 type of health and fitness–related social media content. Predictors of consuming at least one type of health and fitness–related social media content in univariable analysis included female gender (OR 3.5, 95% CI 2.5-4.9, *P*<.001), being aged 15-17 years (OR 3.0, 95% CI 2.2-4.0, *P*<.001), residing outside a major city (OR 2.0, 95% CI 1.4-2.9, *P*<.001), having no post–high school education (OR 2.2, 95% CI 1.7-2.9, *P*<.001), being born in Australia (OR 2.0, 95% CI 1.2-3.2, *P*=.006), having a self-reported eating disorder (OR 2.4, 95% CI 1.5-3.9, *P*<.001), being a victim of bullying (OR 1.7, CI 1.3-2.3, *P*<.001), misusing detox/laxative teas or diet pills (OR 4.6, 95% CI 2.8-7.6, *P*<.001), never using illegal drugs (OR 1.6, 95% CI 1.2-2.0, *P*=.001), and not engaging in risky single occasion drinking on a weekly basis (OR 2.0, 95% CI 1.3-3.0, *P*=.003).

**Conclusions:**

Consumers of health and fitness–related social media content were predominantly teenaged girls. There is a need to ensure that this social media content portrays responsible health messages and to research further the role of fitspiration pages, detox pages, and diet/fitness plan pages in influencing body image and health behaviors.

## Introduction

### Background

**Figure 1:**
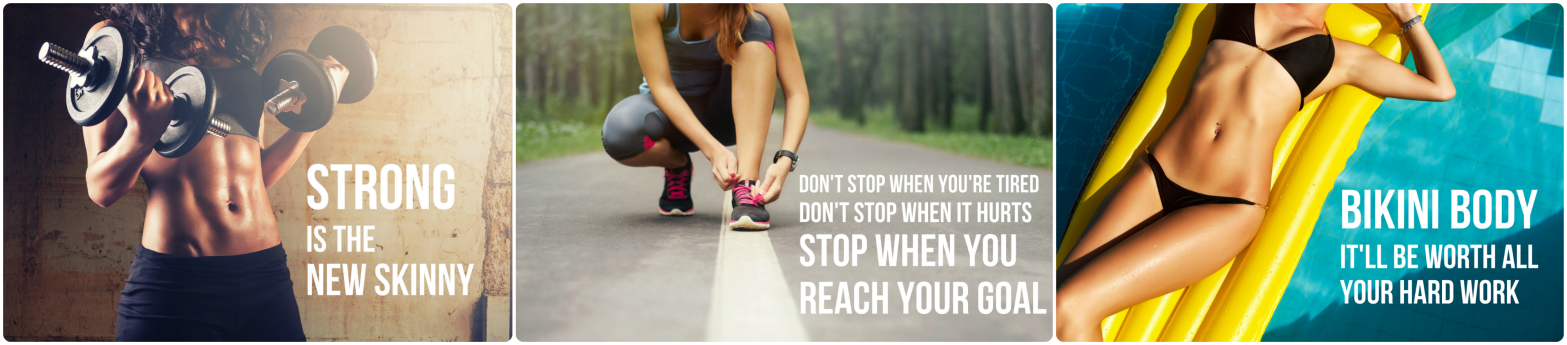
Examples of fitspiration-style images. Photo credit: Shutterstock.

Social media is widely used and accepted among young people. In the United States, up to 90% of teenagers and young adults report using Facebook, whereas more than half use Instagram and one-third use Twitter [1,2]. Young people are increasingly turning to social media as a source of health-related information [3]. A plethora of health and fitness-related social media content is available to young people and is popular, diverse, and interactive; when social media users “like” or “follow” health and fitness-related social media content pages, content appears in their newsfeeds where the user can view and engage with the content by commenting on photos or sharing with friends (through “tagging” or reposting content). One type of health and fitness-related social media content, “fitspiration,” refers to messages designed to inspire individuals to achieve a health or fitness goal, usually through exercise and dieting [4]. Common forms of fitspiration include images of toned bodies overlaid with quotes designed to motivate viewers (Figure 1), blog entries, and personal stories (eg, “before-and-after” weight loss pictures), and personal profiles of fitness trainers and fitness models. Other forms of health and fitness-related social media content include strict diet/exercise plans and “cleanses” or “detoxes” that claim to have health and weight loss benefits. Health and fitness-related social media content is commonly posted by companies to sell a service or product (eg, personal trainers, gyms, or brands of juice detoxes). Health and fitness-related social media content can also be user-generated and maintained; for example, some social media users commonly post exercise “selfies” (self-portrait photographs), statuses about fitness routines, and images of healthy food desired or prepared by the user [3,5].

Health and fitness-related social media content appears to be a double-edged sword. Social media can play a role in shaping body image through social comparison with others and the maintenance of weight- and appearance-related concerns [6-8]. For example, in an exploratory qualitative study into social media’s influence on health behaviors [3], young American adults (mean age 20.4 years) agreed that seeing exercise tips and instructions, using exercise tracking apps, and viewing weight loss before-and-after pictures and fitness-related quotes can be motivational for improving health behaviors. However, some content, such as friends posting fitness-related selfies with negative captions about their physical appearance (eg, “[I’m] still really fat”) can induce negative feelings of body-related shame in the viewer. Content may be misleading, such as advertisements or fitness programs/products conveying unrealistic goals, and users of social media often wish to look their best for their social network and are selective about the content they post [3].

### Criticisms of Health and Fitness–Related Social Media Content

Although many social media-based health and fitness initiatives and interventions are based on scientific research and are run by qualified teams of health experts [9], some health and fitness-related social media content has been criticized for sending inaccurate or irresponsible health messages, a topic of recent media debate. Common criticisms of fitspiration include the prominence of fitspiration images that champion pushing oneself too far during exercise, focus on appearance rather than fitness, and praise the athletic body type (the “athletic ideal”) [10] (Figure 1). Internalization of the athletic ideal has been associated with increased compulsive exercising and negative mood associated with missing an exercise session [11]. Despite a general focus on the athletic ideal body, fitspiration aimed at women often relies on images of slim or thin female bodies to promote an image of what it means to be healthy, fit, and strong [12]. As such, fitspiration has been compared to “thinspiration” and “pro-ana” (pro-anorexia/eating disorder) content, which idealizes thin bodies (the “thin ideal” [13]) and is designed to motivate viewers to lose weight. Exposure to these websites has been associated with adverse effects, such as negative mood and lowered self-esteem, decreased perceived attractiveness, and increased dieting in experimental studies [14,15]. Meanwhile, more than one-third of young people with eating disorders have reported visiting these sites and learning new weight loss and purging techniques [16].

Forms of health and fitness-related social media content that focus on diet, health, and well-being have also been criticized. For example, 2 popular diet programs—the Paleo Diet and the Sugar Free Diet—have been listed by the British Dietetic Association as two of the “Worst Celebrity Diets” and criticized for being unbalanced and unnecessarily restrictive of food groups [17]. Similarly, liquid-based detox diets that claim to rid the body of toxins (despite no medical evidence indicating this is necessary) have been criticized by the Dietitians Association of Australia and can result in the loss of healthy gut bacteria and electrolytes [18]. Despite these concerns, 42% of American adult social media users have reported that information found via social media would affect health decisions related to diet, exercise, or stress management, and nearly 90% of people aged 18 to 24 years have indicated they would trust medical information found on social media [19].

### Past Research

Two recent analyses have indicated that problematic content is posted regularly online under the guise of health. In the first study, which analyzed 21 “healthy living” blogs (which aim to offer advice and personal experiences regarding health), it was found that approximately half contained content with negative or guilt-inducing messages about food and/or content with stigmatizing language relating to weight [20]. In the second study, the authors compared 50 websites dedicated to fitspiration with 50 thinspiration websites. The authors found that although thinspiration websites were more likely than fitspiration websites to contain content praising thinness (34% vs 10%) and championing weight loss (68% vs 42%), both types of websites were equally as likely to contain objectifying content (32% vs 36%), guilt-inducing messages about weight or the body (both 36%), and stigmatizing messages around fat and weight (both 20%) [4]. The authors suggested that although thinspiration appears more obviously detrimental to viewers’ health and body image, it is within reason to assume that viewing both types of websites may negatively impact viewers [4]. Therefore, fitspiration may also attract people with preexisting eating disorder symptomology or vulnerability, or influence emerging psychological concerns such as orthorexia nervosa, an obsession with healthy eating, food quality, and food “purity” with links to obsessive-compulsive disorder and anorexia [21].

### This Study

Despite significant debate in the media about the potential harms of health and fitness-related social media content [12,17,18], little research has examined this content. Previous research has focused on the content of self-labeled fitspiration rather than expanding the scope of this field of research to observe other types of health and fitness-related social media content. Specifically, it is unclear who is liking and following (“consuming”) health and fitness-related social media content via social media. The overarching aim of this exploratory study was to identify the characteristics of young people who consume 3 types of health and fitness-related social media content: fitspiration pages, detox pages, and diet/fitness plan pages. The secondary aims of this study were to determine (1) which demographics predict consuming health and fitness-related social media content, (2) whether young people who consume health and fitness-related social media content have poorer self-rated mental health than those who do not consume this content, and (3) whether young people who consume health and fitness-related social media content use various legal and illegal substances at different rates compared to those who do not consume this content. It was hypothesized that health and fitness-related social media content would be more commonly consumed by young women versus young men and that self-reported mental health problems and misuse of detox teas/laxatives and diet pills would be associated with consuming health and fitness-related social media content.

## Methods

### Data

Participants were recruited via the 2015 Sex, Drugs and Rock’n’Roll study, developed by Burnet Institute: a cross-sectional convenience sample of people aged 15 to 29 years living in Victoria, Australia. The study consisted of a 112-question online survey, which covered demographics, social media use, and general mental and sexual health. The survey was available on Burnet Institute’s website for 6 weeks between February and March 2015. Participants were recruited via social media, advertisements on Facebook targeted to young people, and word of mouth. Only complete responses were analyzed. Participants had the opportunity to win a gift voucher for participating. Informed consent was obtained from each participant. Approval for this study was granted by the Alfred Hospital Human Research Ethics Committee, Melbourne, Victoria. No specific funding was received for this study.

### Measures

#### Demographics

Demographic details included gender (male, female, transgender, or other with option to specify) and age, which was calculated from month and year of birth. A binary variable was created for gender (male/female; due to sample size, only participants identifying as male and female were included in gender-related data analyses) and age was recoded into 3 categories (15-17, 18-19, and 20-29 years). Country of birth was dichotomized as Australian-born or born outside Australia. Participants specified their highest level of completed education, which informed a binary variable which distinguished between participants who were currently completing or had completed any post–high school education and those with high school education or lower (including those still at high school). Participants indicated their sexual identity; a binary variable distinguished between participants identifying as heterosexual and participants identifying as gay, homosexual, lesbian, bisexual, queer, questioning, or other (GLBQQ+). Recreational spending was analyzed to assess socioeconomic status and dichotomized as less than AUD $120 to spend on oneself per week or AUD $120 or more. Participants’ postcodes informed a binary variable to indicate their area of residence, which was major city or nonmajor city.

#### Health and Fitness–Related Social Media Content

Participants were asked, “Do you like/follow any of the following types of pages on Facebook, Instagram, or Twitter?” with the option to choose all that applied. Four of the options were as follows, based on researcher observations of 3 common types of health and fitness–related social media content and a fourth option that was used for the purpose of comparison:

1. Weight loss/fitness motivation profiles (eg, personal trainers, athletes, fitness models)
2. Cleanses or detoxes (eg, , juice detox)
3. Diet plans or weight loss/fitness challenges (eg, I Quit Sugar, Michelle Bridges 12WBT, Kayla Itsines Bikini Body Challenge)
4. Other health-related pages (eg, Cancer Council)

Binary variables were created to identify whether participants liked or followed (ie, consumed) the 3 types of health and fitness–related social media content of interest (hereafter referred to as fitspiration pages, detox pages, and diet/fitness plan pages) and other health pages, respectively (see Multimedia Appendices 1-3 for screenshot examples). A binary yes/no variable was created to identify whether participants consumed at least 1 of the 3 types of health and fitness–related social media content of interest.

#### Mental Health

Participants were asked, “In the last 6 months have you had any mental health problems? This includes any issues that you haven’t spoken to a health professional about.” Options were “yes,” “no,” or “I don’t wish to say.” If participants answered “yes” to having mental health problems in the last 6 months, they were asked, “Could you please specify what this mental health problem/s was?” with an option to choose all that applied. Options were anxiety disorder (eg, generalized anxiety disorder, obsessive-compulsive disorder), mood disorder (eg, depression, bipolar disorder), eating disorder (eg, anorexia nervosa, bulimia), “I don’t wish to say,” and “other” with the option to specify. Binary yes/no variables were created to identify participants experiencing anxiety disorders, mood disorders, and/or eating disorders. Participants were asked if they had been the victim of bullying in the last 6 months, which informed a binary yes/no variable.

#### Substance Use

Participants were asked to report illegal drug use both in their lifetime and in the last month, if they currently smoked cigarettes, and how often they consumed alcohol. Binary yes/no variables were created for these behaviors; weekly “risky single occasion drinking” was defined as consuming 6 or more standard drinks on a weekly basis based on the Alcohol Use Disorders Identification Test [22]. Participants were also asked, “‘In the last 12 months, have you used any of the following drugs/substances illicitly, not as directed or prescribed to someone else? (Tick all that apply)” with options including diet pills and detox/laxative teas. A binary yes/no variable was created to identify participants who had ever misused either diet pills or detox/laxative teas.

### Analysis

All statistical analyses were performed using Stata version 13 (StataCorp LP, College Station, TX, USA). Cross-tabulations and univariable logistic regression were used to compare differences in demographics, mental health, and substance use between those who consumed health and fitness–related social media content (“consumers”) and those who did not. Multivariable logistic regression was performed using variables significant at *P*<.05 at the univariable level to identify independent predictors of consuming health and fitness–related social media content. In the multivariable model, variables significant with the Bonferroni-adjusted *P* value of .0125 (.05/4 tests) were deemed to be significant independent predictors of consuming health and fitness–related social media content.

## Results

The survey was completed by 1001 participants*.* The mean age was 21.40 years (SD 4.12) and the median age was 21.06 years (IQR 17.64-24.74); 269 (26.87%) identified as male, 723 (72.23%) identified as female, 4 (0.40%) identified as transgender, 3 (0.20%) reported their gender as “other,” and 2 (0.20%) did not specify their gender. A total of 308 participants (30.77%) consumed fitspiration pages, 145 (14.49%) consumed detox pages, and 235 (23.48%) consumed diet/fitness plan pages. In all, 378 (37.76%) participants consumed at least 1 of the 3 types of health and fitness–related social media content, 212 (21.17%) consumed at least 2 types, and 96 (9.59%) participants consumed all 3 types. Of the health and fitness–related social media content consumers, 85.7% (324/378) identified as female and 44.8% (324/723) of all female participants consumed at least one type of health and fitness–related social media content. Further, 57.1% (184/322) of teenaged girls consumed at least one type of health and fitness-related social media content; 48.7% (184/378) of all consumers were teenaged girls.

Univariable logistic regression compared health and fitness–related social media content consumers (378/1001, 37.76%) and consumers of other health pages (358/1001, 35.76%). Consuming other health pages predicted consuming health and fitness–related social media content (OR 2.6, 95% CI 2.0-3.4, *P*<.001). Other health pages were significantly more likely to be consumed by female participants than male participants (OR 1.6, 95% CI 1.2-2.1, *P*=.003) and GLBQQ+ participants than heterosexual participants (OR 1.6, 95% CI 1.2-2.1, *P*=.003). No significant results were observed for other demographics regarding other health pages.

Logistic regression was used to examine correlates of consuming at least 1 of the 3 types of health and fitness–related social media content (Table 1). In univariable analysis, significant differences (*P*<.05) were found; consumers of any health and fitness–related social media content were more likely to report female gender, younger age, location in a nonmajor city, no post–high school education, being born in Australia, experiencing eating disorders, being a victim of bullying, misusing detox/laxative teas or diet pills, never using illegal drugs, and not engaging in weekly risky single occasion drinking compared to those who did not consume any health and fitness–related social media content. In multivariable analysis (pseudo *R* ^2^=.11), significant independent predictors of consuming any health and fitness–related social media content at Bonferroni-adjusted *P*<.0125 were female gender (OR 2.6, 95% CI 1.8-3.7, *P*<.001), being aged 15-17 years (OR 2.5, 95% CI 1.4-4.4, *P=*.002), and misusing diet pills or detox teas (OR 3.5, 95% CI 2.0-5.9, *P*<.001).

**Table 1:** Descriptive statistics and univariable logistic regression comparing consumers of at least one type of health and fitness–related social media content (consumers) and participants who did not consume any health and fitness–related social media content (nonconsumers).

Variable		Total, n (%) N=1001	Consumers, n (%) n=378	Nonconsumers, n (%) n=623	OR (95% CI)	*P*
**Gender**						
	Male	269 (26.87)	51 (19.0)	218 (81.0)	1.0	
	Female	723 (72.23)	324 (44.8)	399 (55.2)	3.5 (2.5-4.9)	<.001
**Age (years)**						
	15-17	279 (27.87)	154 (55.2)	125 (44.8)	3.0 (2.2-4.0)	<.001
	18-19	128 (12.79)	51 (39.8)	77 (60.2)	1.6 (1.1-2.4)	.02
	20-29	594 (59.34)	173 (29.1)	421 (70.9)	1.0	
**Place of residence**						
	Nonmajor city	137 (13.69)	72 (52.6)	65 (47.5)	2.0 (1.4-2.9)	<.001
	Major city	846 (84.52)	300 (35.6)	246 (64.5)	1.0	
**Education**						
	No post–high school	336 (33.57)	169 (50.3)	167 (49.7)	2.2 (1.7-2.9)	<.001
	Post–high school	664 (66.33)	208 (31.3)	456 (68.7)	1.0	
**Country of birth**						
	Australia	893 (89.21)	350 (39.2)	543 (60.8)	2.0 (1.2-3.2)	.006
	Outside Australia	97 (9.69)	24 (24.7)	73 (75.3)	1.0	
**Sexual identity**						
	Heterosexual	768 (76.72)	301 (39.2)	467 (60.8)	1.3 (1.0-1.8)	.10
	GLBQQ+^a^	229 (22.88)	76 (33.2)	153 (66.8)	1.0	
**Recreational spending per week (AUD $)**						
	<$120	770 (76.92)	299 (38.8)	471 (61.2)	1.2 (0.9-1.7)	.20
	≥$120	226 (22.58)	77 (34.1)	149 (65.9)	1.0	
**Anxiety** ^b^						
	Yes	404 (40.36)	156 (38.6)	248 (61.4)	1.1 (0.8-1.4)	.640
	No	597 (59.64)	222 (37.2)	375 (62.8)	1.0	
**Eating disorder** ^b^						
	Yes	76 (7.59)	44 (57.9)	32 (42.1)	2.4 (1.5-3.9)	<.001
	No	925 (92.41)	334 (36.1)	591 (63.9)	1.0	
**Mood disorder** ^b^						
	Yes	365 (36.46)	151 (41.4)	214 (58.6)	1.3 (1.0-1.7)	.08
	No	636 (63.54)	227 (35.7)	709 (64.3)	1.0	
**Bullied (last 6 months)**						
	Yes	265 (26.47)	126 (47.6)	139 (52.5)	1.7 (1.3-2.3)	<.001
	No	736 (73.53)	252 (34.2)	484 (65.8)	1.0	
**Misused detox/laxative teas or diet pills (last 12 months)**						
	Yes	83 (8.29)	59 (71.1)	24 (28.9)	4.6 (2.8-7.6)	<.001
	No	918 (91.71)	319 (34.8)	599 (65.3)	1.0	
**Ever used illegal drugs**						
	Yes	545 (54.45)	180 (33.0)	365 (67.0)	1.0	
	No	446 (44.56)	195 (43.7)	257 (56.3)	1.6 (1.2-2.0)	.001
**Last month illegal drug use if ever used drugs**						
	Yes	330 (33.00)	111 (33.6)	219 (66.4)	1.1 (0.7-1.5)	.71
	No	215 (21.48)	69 (32.1)	146 (67.9)	1.0	
**Risky single occasion drinking weekly or more often**						
	Yes	122 (12.19)	30 (24.6)	92 (75.4)	1.0	
	No	758 (75.72)	295 (38.9)	61.1 (463)	2.0 (1.3-3.0)	.003
**Current smoker**						
	Yes	206 (20.68)	71 (34.6)	135 (65.6)	1.0	
	No	790 (79.32)	304 (38.5)	486 (61.5)	1.2 (0.9-1.6)	.290

^a^ Gay, lesbian, bisexual, queer, or questioning.

^b^ Based on self-reported diagnosed and undiagnosed conditions in the last 6 months.

Univariable analyses were repeated for each type of health and fitness–related social media content separately (Tables 2-4). In multivariable regression (pseudo *R* ^2^=.09), significant independent predictors of consuming fitspiration pages at Bonferroni-adjusted *P*<.0125 were female gender (OR 2.0, 95% CI 1.4-2.8, *P*<.001), being aged 15-17 years (OR 2.7, 95% CI 1.5-4.9, *P=*.002), identifying as heterosexual (OR 1.6, 95% CI 1.1-2.4, *P=*.009), and misusing diet pills or detox teas (OR 2.1, 95% CI 1.3-3.5, *P*=.004). Significant independent predictors of consuming detox pages at Bonferroni-adjusted *P*=.01 in multivariable regression (pseudo *R* ^2^=.24) were female gender (OR 52.1, 95% CI 7.2-377.6, *P*<.001), being aged 15-17 years (OR 3.4, 95% CI 1.5-7.7, *P=*.003), misusing diet pills or detox teas (OR 4.7, 95% CI 2.7-8.0, *P*<.001) and using illegal drugs in the last month (OR 2.5, 95% CI 1.3-4.9, *P=*.008). Significant independent predictors of consuming diet/fitness plan pages at Bonferroni-adjusted *P*<.0125 in multivariable regression (pseudo *R* ^2^=.17) were female gender (OR 9.8, 95% CI 4.9-19.6, *P*<.001) and misusing diet pills or detox teas (OR 3.6, 95% CI 2.2-6.0, *P*<.001).

**Table 2:** Descriptive statistics and univariable logistic comparing consumers of fitspiration pages and nonconsumers.

Variable		Total, n (%) N=1001	Consumers, n (%) n=308	Nonconsumers, n (%) n=693	OR (95% CI)	*P*
**Gender**						
	Male	269 (26.87)	48 (17.8)	221 (82.2)	1.0	
	Female	723 (72.23)	259 (35.8)	464 (64.2)	2.6 (1.8-3.6)	<.001
**Age (years)**						
	15-17	279 (27.87)	128 (45.9)	151 (54.1)	2.8 (2.1-3.8)	<.001
	18-19	128 (12.79)	43 (33.6)	85 (66.4)	1.7 (1.1-2.6)	.01
	20-29	594 (59.34)	137 (23.1)	457 (76.9)	1.0	
**Location**						
	Nonmajor city	137 (13.69)	60 (43.8)	77 (65.2)	1.9 (1.3-2.8)	<.001
	Major city	846 (84.52)	243 (28.7)	603 (71.3)	1.0	
**Education**						
	No post–high school	336 (33.57)	138 (41.1)	198 (58.9)	2.0 (1.5-2.7)	<.001
	Post–high-school	664 (66.33)	169 (25.5)	495 (74.6)	1.0	
**Country of birth**						
	Australia	893 (89.21)	286 (32.0)	607 (68.0)	1.8 (1.1-3.0)	.02
	Outside Australia	97 (9.69)	20 (20.6)	77 (79.4)	1.0	
**Sexual identity**						
	Heterosexual	768 (76.72)	251 (32.7)	517 (67.3)	1.5 (1.1-2.1)	.02
	GLBQQ+^a^	229 (22.88)	56 (24.5)	173 (75.6)	1.0	
**Recreational spending per week (AUD $)**						
	<$120	770 (76.92)	248 (32.3)	522 (67.8)	1.3 (1.0-1.9)	
	≥$120	226 (22.58)	59 (26.1)	167 (73.9)	1.0	.08
**Anxiety** ^b^						
	Yes	404 (40.36)	125 (30.9)	279 (69.1)	1.0 (0.8-1.3)	.92
	No	597 (59.64)	183 (30.7)	414 (69.3)	1.0	
**Eating disorder** ^b^						
	Yes	76 (7.59)	38 (50.0)	38 (50.0)	2.4 (1.5-3.9)	<.001
	No	925 (92.41)	270 (29.2)	655 (70.8)	1.0	
**Mood disorder** ^b^						
	Yes	365 (36.46)	124 (34.0)	241 (66.0)	1.3 (1.0-1.7)	.10
	No	636 (63.54)	184 (28.9)	452 (71.1)	1.0	
**Bullied (last 6 months)**						
	Yes	265 (26.47)	100 (37.7)	165 (62.3)	1.5 (1.1-2.1)	.004
	No	736 (73.53)	208 (28.3)	528 (71.7)	1.0	
**Misused detox/laxative teas or diet pills (last 12 months)**						
	Yes	83 (8.29)	44 (53.0)	39 (47.0)	2.8 (1.8-4.4)	<.001
	No	918 (91.71)	264 (28.8)	654 (71.2)	1.0	
**Ever used illegal drugs**						
	Yes	545 (54.45)	140 (25.7)	405 (74.3)	1.0	
	No	446 (44.56)	166 (37.2)	280 (62.8)	1.7 (1.3-2.3)	<.001
**Last month illegal drug use if ever used drugs**						
	Yes	330 (33.00)	84 (25.5)	246 (74.6)	0.9 (0.7-1.4)	.88
	No	215 (21.48)	56 (26.0)	159 (74.0)	1.0	
**Risky single occasion drinking weekly or more often**						
	Yes	122 (12.19)	240 (31.7)	518 (68.3)	1.0	
	No	758 (75.72)	21 (17.2)	191 (82.8)	2.2 (1.4-3.7)	.001
**Current smoker**						
	Yes	206 (20.68)	58 (28.1)	148 (71.8)	1.0	
	No	790 (79.32)	247 (31.3)	543 (68.7)	1.2 (0.8-1.6)	.39

^a^ Gay, lesbian, bisexual, queer, or questioning.

^b^ Based on self-reported diagnosed and undiagnosed conditions in the last 6 months.

**Table 3:** Descriptive statistics and univariable logistic comparing consumers of detox pages and nonconsumers.

Variable		Total, n (%) N=1001	Consumers, n (%) n=145	Nonconsumers, n (%) n=856	OR (95% CI)	*P*
**Gender**						
	Male	269 (26.87)	1 (0.4)	268 (99.6)	1.0	
	Female	723 (72.23)	142 (19.6)	581 (80.4)	65.5 (9.1-470.7)	<.001
**Age (years)**						
	15-17	279 (27.87)	81 (29.0)	198 (71.0)	5.2 (3.5-7.9)	<.001
	18-19	128 (12.79)	21 (16.4)	107 (83.6)	2.5 (1.4-4.4)	.001
	20-29	594 (59.34)	43 (7.2)	551 (92.8)	1.0	
**Location**						
	Nonmajor city	137 (13.69)	36 (26.3)	101 (73.7)	2.4 (1.6-3.7)	<.001
	Major city	846 (84.52)	108 (12.8)	738 (87.2)	1.0	
**Education**						
	No post–high school	336 (33.57)	89 (26.5)	247 (73.5)	3.9 (2.7-5.6)	<.001
	Post–high school	664 (66.33)	56 (8.4)	608 (91.6)	1.0	
**Country of birth**						
	Australia	893 (89.21)	136 (15.2)	757 (84.8)	2.0 (0.9-4.2)	.07
	Outside Australia	97 (9.69)	8 (8.3)	89 (91.8)	1.0	
**Sexual identity**						
	Heterosexual	768 (76.72)	118 (15.4)	650 (84.6)	1.4 (0.9-2.2)	.13
	GLBQQ+^a^	229 (22.88)	26 (11.4)	203 (88.7)	1.0	
**Recreational spending per week (AUD $)**						
	<$120	770 (76.92)	122 (15.8)	648 (84.2)	1.7 (1.1-2.8)	.02
	≥$120	226 (22.58)	22 (9.7)	204 (90.3)	1.0	
**Anxiety** ^b^						
	Yes	404 (40.36)	67 (16.6)	337 (83.4)	1.3 (0.9-1.9)	.12
	No	597 (59.64)	78 (13.1)	519 (86.9)	1.0	
**Eating disorder** ^b^						
	Yes	76 (7.59)	25 (32.9)	51 (67.1)	3.3 (2.0-5.5)	<.001
	No	925 (92.41)	120 (13.0)	805 (87.0)	1.0	
**Mood disorder** ^b^						
	Yes	365 (36.46)	63 (17.3)	302 (82.7)	1.4 (1.0-2.0)	.06
	No	636 (63.54)	82 (12.9)	553 (87.1)	1.0	
**Bullied (last 6 months)**						
	Yes	265 (26.47)	51 (19.3)	214 (80.8)	1.6 (1.1-2.4)	.01
	No	736 (73.53)	94 (12.8)	642 (87.2)	1.0	
**Misused detox/laxative teas or diet pills (last 12 months)**						
	Yes	83 (8.29)	40 (48.2)	43 (51.8)	7.2 (4.5-11.6)	<.001
	No	918 (91.71)	105 (11.4)	813 (88.6)	1.0	
**Ever used illegal drugs**						
	Yes	545 (54.45)	72 (13.2)	473 (86.8)	1.0	
	No	446 (44.56)	72 (16.1)	374 (83.9)	1.3 (0.9-1.8)	.19
**Last month illegal drug use if ever used drugs**						
	Yes	330 (33.00)	57 (17.3)	273 (82.7)	2.8 (1.5-5.1)	.001
	No	215 (21.48)	15 (7.0)	200 (93.0)	1.0	
**Risky single occasion drinking weekly or more often**						
	Yes	122 (12.19)	12 (9.8)	110 (90.2)	1.0	
	No	758 (75.72)	112 (14.8)	646 (85.2)	1.6 (0.9-3.0)	.15
**Current smoker**						
	Yes	206 (20.68)	37 (18.0)	169 (82.0)	1.4 (0.9-2.1)	.10
	No	790 (79.32)	106 (13.4)	684 (86.6)	1.0	

^a^ Gay, lesbian, bisexual, queer, or questioning.

^b^ Based on self-reported diagnosed and undiagnosed conditions in the last 6 months.

**Table 4:** Descriptive statistics and univariable logistic comparing consumers of diet/fitness plan pages and nonconsumers.

Variable		Total, n (%) N=1001	Consumers, n (%) n=235	Nonconsumers, n (%) n=766	OR (95% CI)	*P*
**Gender**						
	Male	269 (26.87)	9 (3.4)	260 (96.7)	1.0	
	Female	723 (72.23)	225 (31.1)	498 (68.9)	13.0 (6.6-25.8)	<.001
**Age (years)**						
	15-17	279 (27.87)	106 (38.0)	173 (62.0)	3.2 (2.3-4.4)	<.001
	18-19	128 (12.79)	33 (25.8)	95 (74.2)	1.8 (1.1-2.8)	.01
	20-29	594 (59.34)	96 (16.2)	498 (83.8)	1.0	
**Location**						
	Nonmajor city	137 (13.69)	40 (29.2)	97 (70.8)	1.4 (0.9-2.1)	.09
	Major city	846 (84.52)	191 (22.6)	655 (77.4)	1.0	
**Education**						
	No post–high school	336 (33.57)	118 (35.1)	218 (64.9)	2.5 (1.9-3.4)	<.001
	Post–high school	664 (66.33)	117 (17.6)	547 (82.4)	1.0	
**Country of birth**						
	Australia	893 (89.21)	221 (24.8)	672 (75.3)	2.3 (1.2-4.3)	.008
	Outside Australia	97 (9.69)	12 (12.4)	85 (87.6)	1.0	
**Sexual identity**						
	Heterosexual	768 (76.72)	189 (24.6)	579 (75.4)	1.3 (0.9-1.9)	.12
	GLBQQ+^a^	229 (22.88)	45 (19.7)	184 (80.4)	1.0	
**Recreational spending per week (AUD $)**						
	<$120	770 (76.92)	190 (24.7)	580 (75.3)	1.4 (1.0-2.0)	.08
	≥$120	226 (22.58)	43 (19.0)	183 (81.0)	1.0	
**Anxiety** ^b^						
	Yes	404 (40.36)	107 (26.5)	297 (73.5)	1.3 (1.0-1.8)	.07
	No	597 (59.64)	128 (21.4)	469 (78.6)	1.0	
**Eating disorder** ^b^						
	Yes	76 (7.59)	35 (46.0)	41 (54.0)	3.1 (1.9-5.0)	<.001
	No	925 (92.41)	200 (31.6)	725 (78.4)	1.0	
**Mood disorder** ^b^						
	Yes	365 (36.46)	101 (27.7)	264 (72.3)	1.4 (1.1-1.9)	.02
	No	636 (63.54)	134 (21.1)	502 (78.9)	1.0	
**Bullied (last 6 months)**						
	Yes	265 (26.47)	91 (34.3)	174 (65.7)	2.2 (1.6-2.9)	<.001
	No	736 (73.53)	144 (19.6)	592 (80.4)	1.0	
**Misused detox/laxative teas or diet pills (last 12 months)**						
	Yes	83 (8.29)	48 (57.8)	35 (42.2)	5.3 (3.4-8.5)	<.001
	No	918 (91.71)	187 (20.4)	731 (79.6)	1.0	
**Ever used illegal drugs**						
	Yes	545 (54.45)	107 (19.6)	438 (80.4)	1.0	
	No	446 (44.56)	126 (28.3)	320 (71.8)	1.6 (1.2-2.2)	.002
**Last month illegal drug use if ever used drugs**						
	Yes	330 (33.00)	72 (21.8)	258 (78.2)	1.4 (0.9-2.2)	.11
	No	215 (21.48)	35 (16.3)	180 (83.7)	1.0	
**Risky single occasion drinking weekly or more often**						
	Yes	122 (12.19)	21 (17.2)	101 (82.8)	1.0	
	No	758 (75.72)	179 (23.6)	579 (76.4)	1.5 (0.9-2.4)	.12
**Current smoker**						
	Yes	206 (20.68)	47 (22.8)	159 (77.2)	1.0 (0.7-1.5)	.83
	No	790 (79.32)	186 (23.5)	604 (46.5)	1.0	

^a^ Gay, lesbian, bisexual, queer, or questioning.

^b^ Based on self-reported diagnosed and undiagnosed conditions in the last 6 months.

## Discussion

Our study, to the best of our knowledge, was the first to explore characteristics of the consumers of health and fitness-related social media content. Our results indicate that consuming health and fitness-related social media content is common; 378 of 1001 (37.76%) participants reported liking or following at least one of the included health and fitness-related social media content types on Facebook, Instagram, or Twitter, most commonly fitspiration pages (308/1001, 30.8%), followed by diet/fitness plan pages (235/1001, 23.5%), and detox pages (145/14.49, 14.5%). The majority of health and fitness-related social media content consumers identified as female, supporting our hypothesis. This result was unsurprising; health and fitness-related social media content is largely aimed at women and often driven by female celebrities and fitness models. Considering the number of objectifying messages previously observed in fitspiration [4], and the potential internalization of messages such as these by girls and women in Western society [23], it is potentially concerning that nearly half of female participants reported consuming this content. Even so, some health and fitness-related social media content is aimed at men (ie, bodybuilding pages featuring endorsement from male athletes) and there is potential for this content to negatively affect the body image of young men, such as increasing a drive for muscularity [24].

Other demographic differences were noted fairly consistently in the data. Key characteristics of health and fitness-related social media content consumers were being younger and less educated; more than half of participants aged between 15 and 17 years and more than half of participants with no post-high school education (which included those still in high school) reported consuming at least one of the health and fitness-related social media content types, although this latter variable was not significant in adjusted analyses. In all, nearly half of all consumers (48.7%, 184/378) were teenaged girls. These findings are of concern because adolescence is a particularly challenging time in terms of body image [25] and more educated people are generally more likely to engage in healthy behaviors, such as engaging in physical activity and not smoking [26], and have higher health literacy [27].

Some differences were observed regarding mental health and substance use, partially supporting our hypothesis. Participants with eating disorders were 2 to 3 times more likely to consume health and fitness-related social media content than participants without eating disorders. It is likely that this relationship is bidirectional; this content may attract people with eating disorders or body image concerns, but the content may exacerbate or validate symptomology and behaviors [8]. Further, a significant difference emerged with regards to mood disorders: participants with mood disorders were more likely than those without mood disorders to consume diet/fitness plan pages, although this was not significant in adjusted analyses. This finding is interesting in the context of thinspiration research, which has found more negative affect after viewing thinspiration websites [14]. It is unclear why this relationship emerged for the diet/fitness plan pages, but not the other types of health and fitness-related social media content.

Approximately 70% of participants who reported misusing detox/laxative teas or diet pills in the last 6 months consumed any health and fitness-related social media content, supporting our hypothesis. These weight loss materials have been shown to have detrimental health effects and use actually predicts weight gain over time in adolescents [28,29]. Consumers of any health and fitness-related social media content were significantly less likely than nonconsumers to report ever using illegal drugs or to report weekly risky single occasion drinking. This was an interesting finding; it is possible that consumers of health and fitness-related social media content are concerned about the effect of substance use on their health, thereby avoiding consumption. However, several of these results were not significant in adjusted analyses and it is possible that these findings were related to the age of participants because younger participants were more likely to consume health and fitness-related social media content. In adjusted analyses, consumers of detox pages were more likely to have used illegal drugs recently than nonconsumers. This association may be related to use of illegal drugs for weight loss or maintenance, such as psychostimulants [30]. These relationships should be explored in future research.

It is possible that for the majority of consumers, health and fitness-related social media content is beneficial and provides motivation for healthy behaviors. Yet the key characteristics of consumers of health and fitness-related social media content appear to be female gender and a younger age, with at-risk groups including those with eating disorder symptomology, being a victim of bullying, and misusing detox/laxative teas and diet pills. Health and fitness-related social media content potentially has the power to impact on these individuals, including influencing the formation of young people’s norms regarding ideal body shape and what it means to be healthy; emerging research indicates that adolescent girls are increasingly turning to fitness models as role models [31]. It can be difficult to distinguish between health and fitness-related social media content that is helpful or motivational and content that is harmful, and to whom messages championed by this content are negatively affecting; however, it appears that some vulnerable individuals are consuming health and fitness-related social media content.

Nearly 90% of American young adults have reported they would trust medical information found on social media [19]. Therefore, there is a need to ensure that health and fitness-related social media content portrays adequate, responsible health messages championing accurate information about health and fitness, motivating individuals to exercise without shaming those who do not or cannot, having realistic health and fitness goals, and encouraging a healthy lifestyle without objectifying messages. Health promotion initiatives should target consumers of health and fitness-related social media content in terms of health literacy and body positivity, teaching at-risk individuals to be critical of media messages in relation to what it means to be fit and healthy. Some recent campaigns have attempted this, such as the UK campaign “This Girl Can,” which aims to celebrate women’s participation in sport regardless of physical appearance [32].

Another possible option for dealing with potential harms of health and fitness-related social media content is regulating social media content, although this can be challenging. Thinspiration is recognized as harmful by most social media sites and is shut down or censored with varying degrees of success [33]; for example, Facebook community standards state “We prohibit content that promotes or encourages...eating disorders” [34] and if users search for thinspiration and related terms, Instagram and Tumblr provide warnings for graphic content and referrals to eating disorder information and recovery resources. However, due to varying rates of effective moderation and social media sites not wishing to censor users’ recovery journeys, it is still easy to find thinspiration content across nearly every social media platform [35]. No specific guidelines exist for health and fitness-related social media content. Current advertising guidelines on Facebook indicate that images that “emphasize an ‘ideal’ body or body parts, or images showing unexpected or unlikely results, such as ‘before and after images’” are not allowed, and that “ads that promote acceptable dietary and herbal supplements may only target users who are at least 18 years of age” [35], but such guidelines do not appear to exist for page or user-generated content, even when the page is advertising a product. Considering problematic health and fitness-related social media content messages may be subtle or labeled as “healthy,” these guidelines may not adequately identify harmful content.

Two additional barriers to regulating health and fitness-related social media content on social media have been raised. Firstly, social media is saturated with health and fitness-related social media content: this content has huge followings (eg, more than 23 million posts on Instagram have been tagged with “#fitspo” at the time of writing) and the nature of social media means that health and fitness-related social media content is often viewed by social media users even if they do not necessarily wish to view it (ie, if one user “likes” an image, this image will then appear in the newsfeeds of many of their friends). The nature of targeted advertisements means that merely mentioning food or exercise on social media can result in users being presented with advertisements related to health and fitness-related social media content [5]. Secondly, health and fitness-related social media content is largely celebrated, user-generated, and talked about in a positive manner, reinforcing content and behaviors to peers on social media [3]. An argument for clinically distinguishing orthorexia from anorexia and obsessive-compulsive disorder is that people with orthorexia are likely to flaunt their health behaviors, such as via social media [21], because these behaviors are largely socially desirable and celebrated, making it difficult to determine where health behaviors are obsessive and/or no longer healthy. The saturation and popularity of health and fitness-related social media content means that its messages are unavoidable for many users of social media and easily normalized regardless of actual health benefits. This reinforces the importance of media literacy and education programs around health and fitness for young people.

The authors recognize the limitations of this study. The sample was an online convenience sample and may not be generalizable to all social media users. The questions asked were broad and lacked specificity; we did not enquire after the number of health and fitness-related social media content pages liked or followed, the degree of interaction with the content, or break down the pages any further (eg, by examining participants who specifically followed self-labeled fitspiration pages on social media). Data were self-reported and, therefore, vulnerable to recall bias; social media users often follow a large number of pages [36] and are unlikely to remember all of them. Those who consumed fitspiration pages, detox pages, and diet/fitness plan pages were more likely to follow other health pages too, possibly reflecting an interest in health in general rather than just the 3 types of health and fitness-related social media content we studied. We only focused on Facebook, Instagram, or Twitter, potentially excluding participants who follow health and fitness-related social media content on Tumblr or Pinterest (used by 23% and 33% of teenage girls, respectively, more than 3 times the rate of use by teenaged boys [2]) or engage with other user-generated forums and groups such as those on Reddit. We only asked about participants misusing diet pills and detox/laxative teas; it would have been worth exploring any use of diet/weight loss materials. Our cross-sectional design and analysis strategy was unable to determine direction of relationships or causality. Due to the length of the larger survey, we did not examine body image or use validated measures of mental health, although single-item self-report measures of psychosocial variables can be as valid as multiple-item scales [37]. Further, due to the exploratory nature of the study, we included a large number of statistical tests; we applied the conservative Bonferroni correction, which thereby increases the risk of type II error.

Observed gender differences are likely related to the types of health and fitness–related social media content we chose to examine. The diet plans and challenges included as examples were heavily female-led (eg, branded with female celebrities) and focused (eg, bikini body challenges), potentially biasing recall. Gender differences may have also been related to women consuming more health-related pages on social media than men in general. We did not specifically examine, or include as examples, health and fitness–related social media content aimed at men, such as bodybuilding or other muscularity-based initiatives. Such health and fitness–related social media content is worth exploring in the future.

This is the first study to characterize consumers of 3 types of health and fitness-related social media content: fitspiration pages, detox pages, and diet/fitness plan pages. Overall, the results of this exploratory study indicate that the consumers of health and fitness-related social media content are largely teenaged girls and that individuals reporting eating disorders and detox or laxative misuse are more likely to consume health and fitness-related social media content. The results emphasize the need to perform further research into this area and consider the role of health and fitness-related social media content in the formation of body image, health ideals and behaviors, and emerging mental health issues such as orthorexia within the complex context of normative processes and development, particularly among at-risk individuals [8]. Future experimental or longitudinal research should determine whether health and fitness-related social media content actually impacts the consumer’s body image and health behaviors and, if so, how it can be addressed. There is also a need to perform a content analysis on health and fitness-related social media content to determine to what degree these pages are championing accurate versus unhealthy or unscientific health messages and determine which social media platforms are best to target for future interventions.

## Appendix

Multimedia Appendix 1 Example of fitspiration page: ‘Fitspore’ profile on Instagram.

Multimedia Appendix 2 Example of detox page: ‘SkinnyMe Tea’ on Facebook.

Multimedia Appendix 3 Example of diet/fitness plan page: ‘Ashy Bines Bikini Body Challenge’ on Facebook.

## Abbreviations

GLBQQ+: gay, lesbian, bisexual, queer, or questioning
