# Supplementary figures and images for: Predictors of “Liking” Three Types of Health and Fitness-Related Content on Social Media: A Cross-Sectional Study
Source: J Med Internet Res. 2015 Aug 21;17(8):e205. doi: 10.2196/jmir.4803 (PMC4642410; doi:10.2196/jmir.4803)

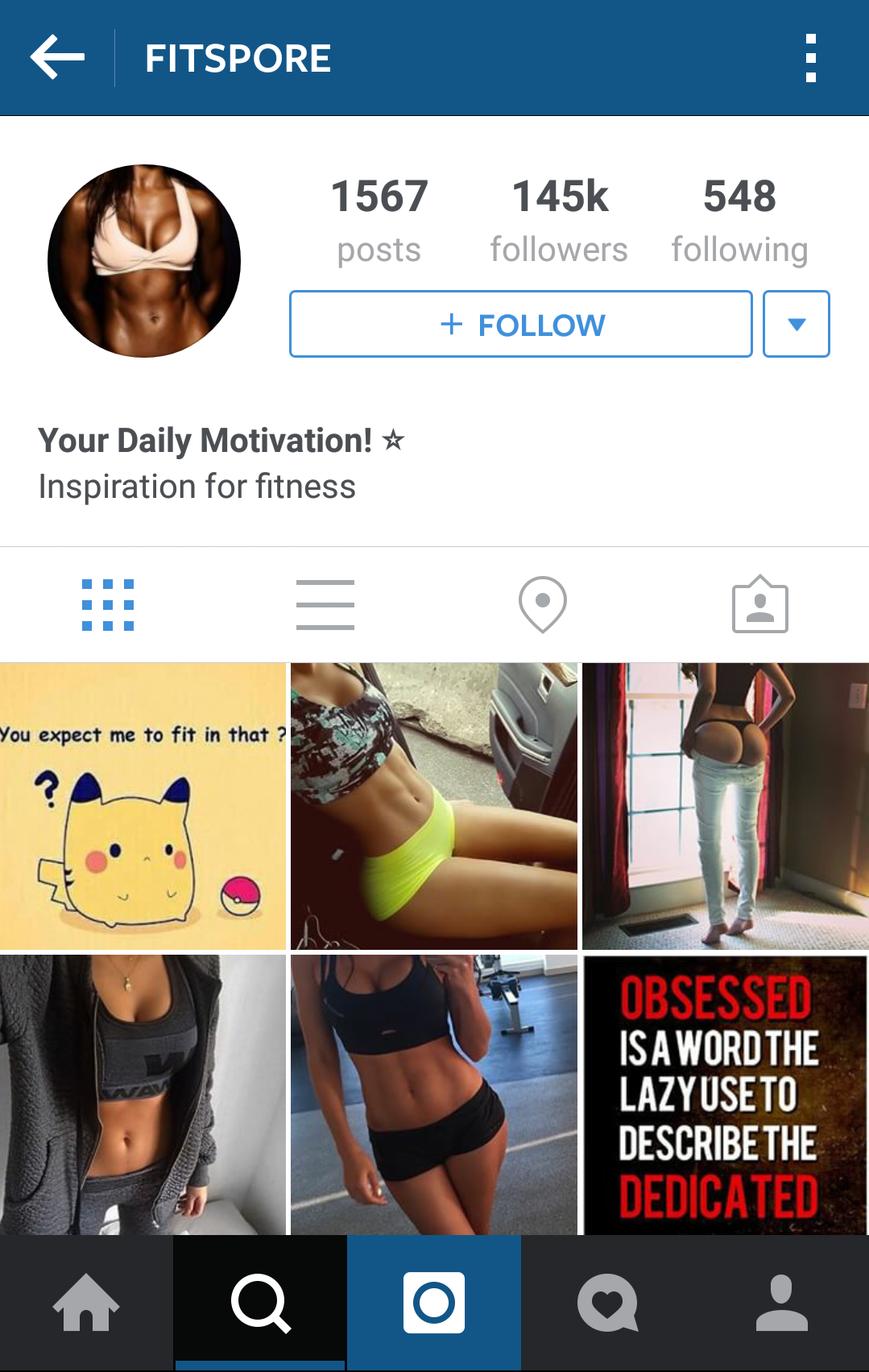

Supplement: Multimedia Appendix 1 [file jmir_v17i8e205_app1.png]

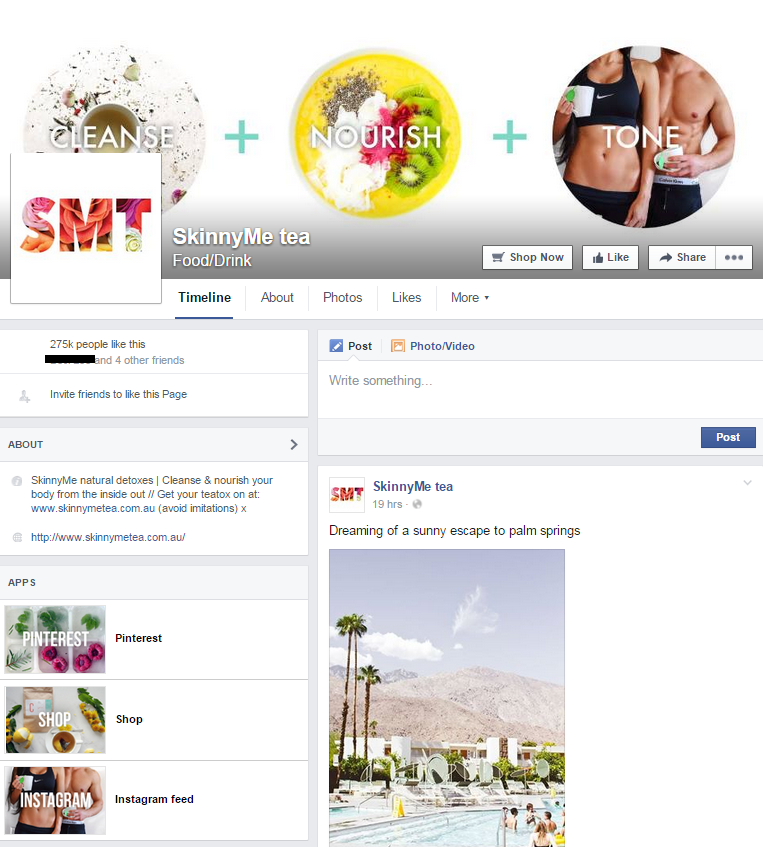

Supplement: Multimedia Appendix 2 [file jmir_v17i8e205_app2.png]

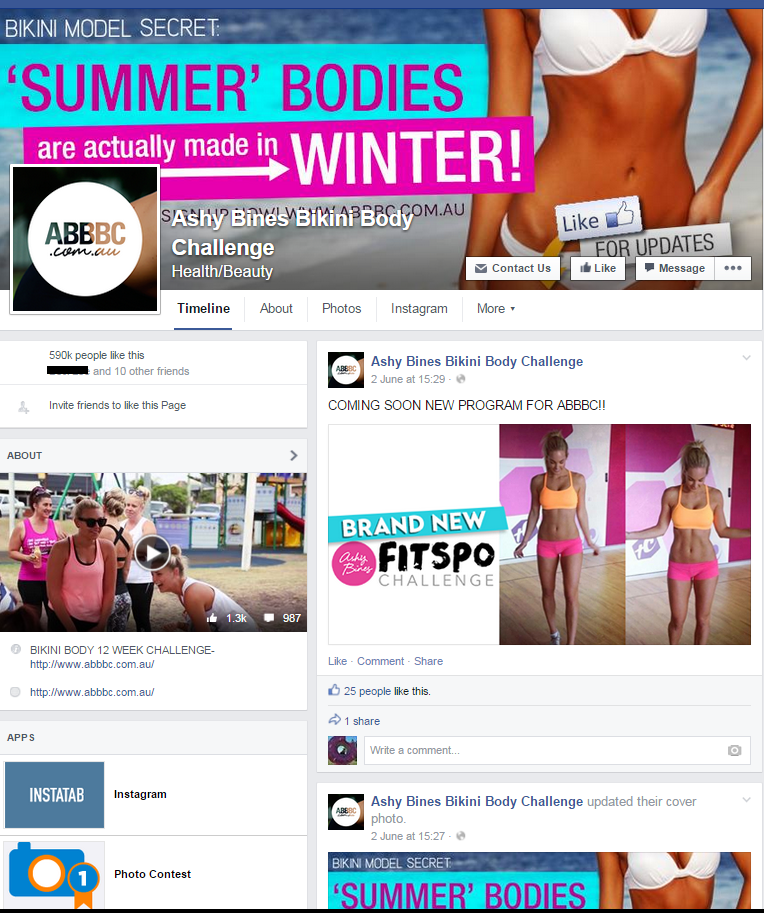

Supplement: Multimedia Appendix 3 [file jmir_v17i8e205_app3.png]
